# Dental Challenges and the Needs of the Population during the Covid-19 Pandemic Period. Real-Time Surveillance Using Google Trends

**DOI:** 10.3390/ijerph17238999

**Published:** 2020-12-03

**Authors:** Magdalena Sycinska-Dziarnowska, Iwona Paradowska-Stankiewicz

**Affiliations:** 1 Individual Dental Practice, 70734 Szczecin, Poland; magdadziarnowska@gmail.com; 2 Department of Epidemiology of Infectious Diseases and Surveillance, National Institute of Public Health—National Institute of Hygiene, 00791 Warsaw, Poland

**Keywords:** COVID-19, SARS-CoV-2, dental treatment needs, oral health, toothache, oral cancer, oral epidemiology, oral prevention, teledentistry

## Abstract

Background: The outbreak of the COVID-19 pandemic may lead to changes in the dental needs of the population and new challenges concerning oral health care. Methods: The Google Trends tool was used to collect data on the Internet search interest. The investigated material was collected from 1 January 2020 to 23 August 2020. Search terms “toothache”, “dentist” and “stay at home” were retrieved for the whole world as well as for the US, the UK, Poland, Italy and Sweden. Results: During the lockdown, correlation analysis indicates the lowest public interest in the word “dentist” one week preceding the peak for “toothache”, followed by an increase in the word search for “dentist”. On 12 April, worldwide, the maximum of Google Trends Relative Search Volume (RSV) for “toothache” was observed. Conclusion: Decrease in “dentist” queries during lockdown followed by an increase in “toothache” search predicts greater dental needs in the post-pandemic period. The surveillance shows significant changes in queries for dental-related terms during the course of the COVID-19 pandemic. In order to prepare for future pandemic outbreaks teledentistry programs should be taken into consideration.

## 1. Introduction

In 2017, The Global Burden of Disease Study estimated that close to 3.5 billion people worldwide were affected by oral diseases, with caries of permanent teeth being the most common condition. Globally, 2.3 billion people suffered from caries of permanent teeth and about 530 million children suffered from tooth decay of the primary teeth [1]. Moreover, in 2018, there were 355,000 cases of oral cancer all over the world which caused 177,000 deaths [2]. The situation regarding oral health care and the prevention of oral diseases became even more complex when on 11 March 2020 the World Health Organization (WHO) declared the outbreak of the novel coronavirus (SARS-CoV-2) pandemic [3]. The SARS-CoV-2 virus was detected in December 2019 in Wuhan, Hubei Province, China. As of 31 August 2020, more than 25.3 million cases of COVID-19 have been reported, resulting in more than 1,131,000 deaths. However, as reported on 22 October, more than 28 million people have overcome the disease [4].

A decrease in dental visits was noted during the COVID-19 pandemic outbreak. According to Guo et al. [5] at the beginning of the COVID-19 pandemic thirty-eight percent fewer patients visited the dental surgery when compared with an earlier period. The decline in dental visits was due to the almost worldwide control steps and preventive measures with the suspension of businesses, schools and universities to flatten the curve of infections [6]. The SARS-CoV-2 virus may be transmitted directly by inhalation of respiratory secretion droplets, as well as by contact with oral, nasal or eye mucous membranes [7]. In order to reduce the virus spread, people were recommended to wear protective face masks, face shields, gloves and use hand sanitizers. As soon as the WHO gave a statement that the virus was easily transmitted by droplets, dental offices were believed to be possible pathways for virus transmission [8].

In recent years, researchers found that social media platforms such as Instagram, Twitter and Facebook can deliver real-time data about the health and behavior of the population [9,10,11]. The easy access and overwhelming information available to the public played crucial roles in the growing interest in online information gathering [12,13]. The amount of medical information retrieved from the Internet and shared among the public through social media and website have largely increased during the COVID-19 pandemic [14].

Google Trends service analyzes the popularity of internet search queries and can become a useful tool for surveillance across different languages and regions all around the globe. It allows the comparison and measurement of a variety of social behaviors [15,16]. No studies based on Google Trends search query concerning dental needs during the COVID-19 pandemic could be found. Studies concerning different data analysis during other outbreaks of diseases like Ebola [17], Zika virus [18], H1N1 [10] were of concern for scientists. Presently COVID-19 disease transmission routes, outcomes and treatments are also gaining considerable research attention [19,20].

The aim of this study was to analyze, during the COVID-19 pandemic, the Google search interest in inquiries for “dentist” and compare it with questions concerning “toothache”.

## 2. Materials and Methods

The Google Trends tool was used to collect data on Internet search interest among Google search engine users. Owned and created by Google, the Google Trends service was launched on 11 May 2006. In this free and publicly available service, data are available on a daily basis [15,16].

The website presents normalized data about anonymous user searches, that range from 0–100, where 100 means the maximum number of searches in a given period of time. In this study, the investigated material was collected from 1 January 2020 to 23 August 2020. Search terms “toothache”, “dentist” and “stay at home” were retrieved from the whole world as well as for the US, the UK, Poland, Italy and Sweden to show differences between countries. When looking at the whole world and English-speaking countries, the search queries were performed in the English language. Regarding Poland, Italy and Sweden, inquiries were stated in the language of the country. The translation from English into Italian and Swedish was confirmed by Google translator. Polish was the native language for the authors. “Toothache” in Polish is translated as “ból zęba”, in Italian “mal di denti” and “tandvärk” in Swedish. “Dentysta” stands for dentist in Polish, as well as “dentista” in Italian and “tandläkare” in Swedish.

In this study, a descriptive comparison between the search volume of “stay at home” and “dentist” together with the phrase “toothache” was conducted. By 30 March 2020 “stay at home” orders were issued by the majority of states in the US [21]. This phrase also became popular in social media, during the period of COVID-19 pandemic #stayathome hashtag gained almost 18 million posts on Instagram. In this study, the “stay at home” phrase was used to visualize on a chart the period of social distancing; the lockdown and quarantine. “Stay at home” was translated into Polish as “zostań w domu”, Italians used “state a casa” and people in Sweden wrote “stanna hemma” in order to encourage national social distancing. The correlation analysis with the Spearman’s Rho coefficient was performed for the words “toothache” and “dentist”. The IBM SPSS Statistics 25 package was used for this purpose. The level of statistical significance was set at α = 0.05. Daily Google Trends normalized search volume was transferred to the spreadsheet and visualized on the charts. Relative Search Volume (RSV) charts were used to show changes in the period of time of investigated phrases. All data collected for the study are publicly available in Google Trends service [15].

## 3. Results

**Figure 1:**
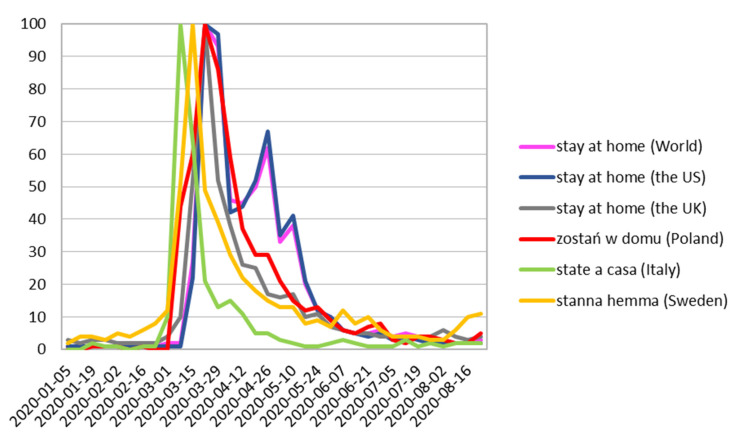
Stay at home phrase Relative Search Volume.

Two peaks of the “stay at home” phrase were observed in both the worldwide and the US searches, compared to single peaks in European countries. The first peak of the “stay at home” phrase was observed worldwide and in the US on 22 March, probably due to the coronavirus orders given by the government [21], and the second peak on the week ending on 26 April. Poland and the UK showed peaks on 22 March while peaks in Italy and Sweden were reported two weeks earlier—on 8 March (Figure 1).

**Figure 2:**
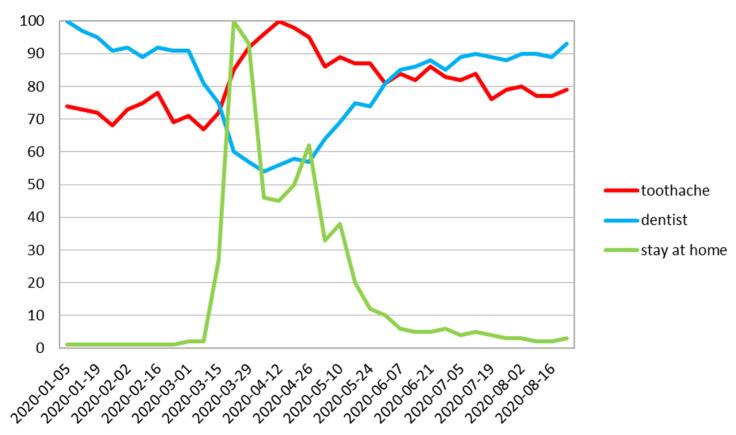
Worldwide relative search volume.

The data for the whole world and the aforementioned countries are summarized in Table 1. Worldwide, a maximum of Google Trends relative search volume (RSV) for “toothache” was observed on 12 April. Statistical analysis indicates that “toothache” and “dentist” queries had significant statistical correlation with r_s_ = −0.76 and *p* < 0.001, which indicates a negative and strong correlation. The negative nature of this relationship means that the fewer inquiries for “dentist” were stated and more searches for “toothache” occurred. The lowest public interest in “dentist” was one week preceding the peak for “toothache” and was followed by an increase of “dentist” searches in June and a decrease in “toothache” queries starting at the end of May (Figure 2).

**Figure 3:**
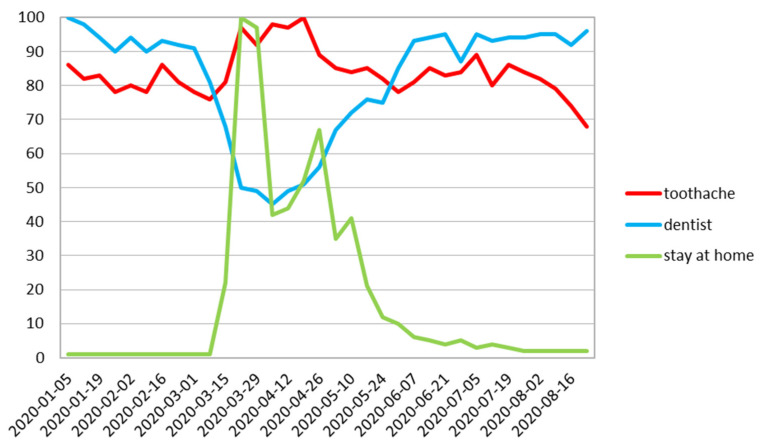
The US relative search volume.

In the US the “toothache” trend imitated the worldwide trend excluding the longer and flatter curve and was postponed by one week. The “Dentist” trend in the US was similar to the worldwide trend, with an even stronger decrease of interest. The moment when there was a decrease in “dentist” and an increase in “toothache” was in line with the worldwide restrictions announced by governments [6]. At the end of the investigated period of time, there was observed a sharper decrease for “toothache” search in the US compared to the worldwide volume (Figure 3).

Differences in the number of search trends are more visible between European countries, as rigorous measures were different among countries.

**Figure 4:**
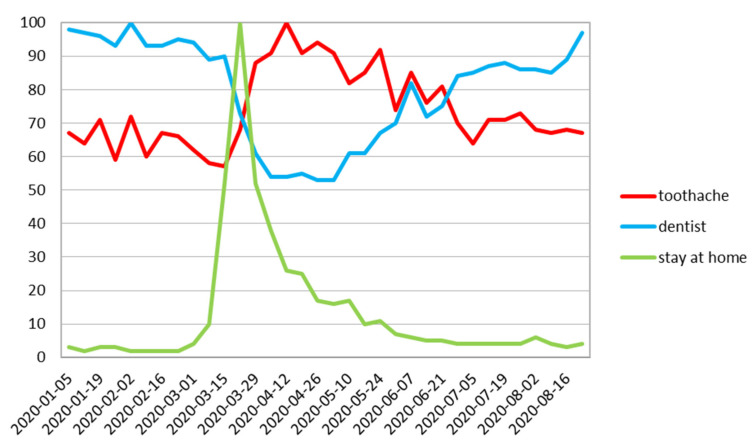
The UK relative search volume.

The UK showed an almost similar pattern to worldwide queries, with an increase in RSV for “toothache” simultaneously followed by a decrease of “dentist” phrase as well as reversed trend starting from the end of June (Figure 4), with higher “dentist” phrase increase in August.

**Figure 5:**
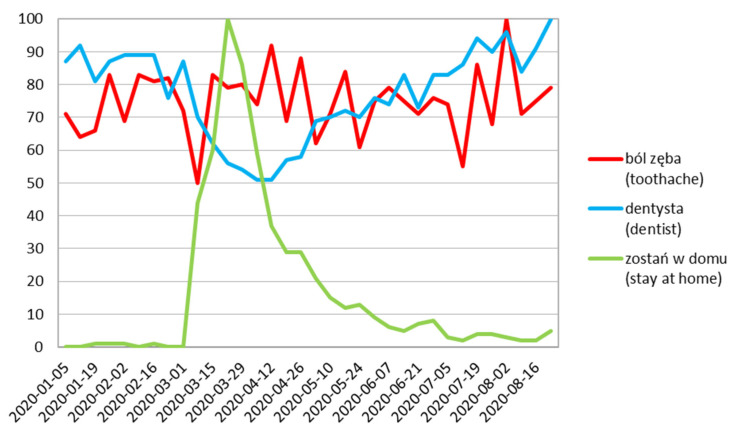
Poland relative search volume.

**Figure 6:**
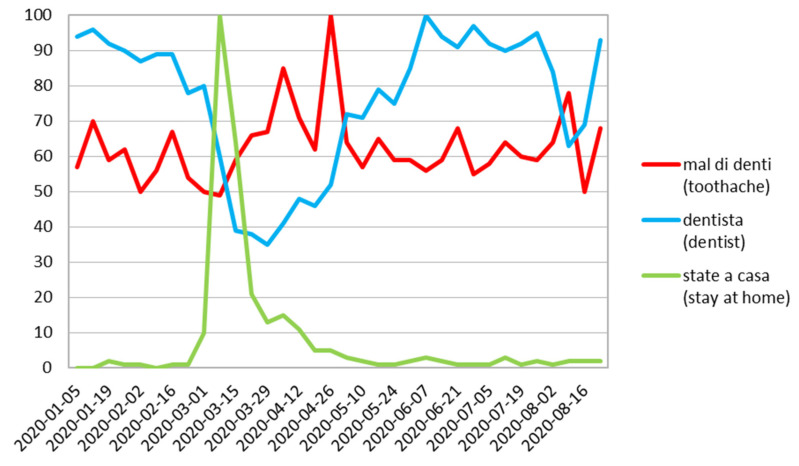
Italy relative search volume.

**Figure 7:**
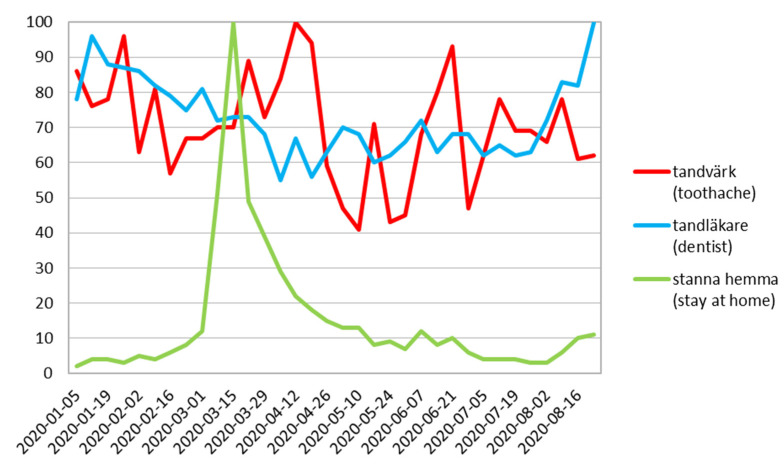
Sweden relative search volume.

Poland, Italy and Sweden show sharper curve changes in the value query for “toothache” (Figure 5, Figure 6 and Figure 7). In Poland, the first big decrease of volume according to the above mentioned was observed on 8 March, with its increase followed by a local peak on 12 April; during the two weeks of lowest queries for “dentist” phrase (Figure 5).

In Italy, two high volumes of search queries for “toothache” were observed: the first peak on the 5th April followed by second peak on 26 April closely followed by a strong decrease in “dentist” observed on 29 March (Figure 6).

On the contrary, in Sweden, the country with the fewest number of restrictions, a sharp decline in interest regarding the word “dentist” was not present. Meanwhile there was one week of larger and another one of smaller index of “toothache” phrase searching (Figure 7).

On all charts in the month of August, the RSV for “dentist” followed a similar path (Figure 2, Figure 3, Figure 4, Figure 5, Figure 6 and Figure 7), with its steadily increasing number of search enquiries for “dentist”.

## 4. Discussion

This is the first study that has used the Google Trends queries concerning the subject of dental treatment needs during COVID-19 pandemic. In our study, we found a decrease in RSV Google Trends for the word “dentist” with a significant increase in the search for “toothache” after a short period of time (Figure 2, Figure 3, Figure 4, Figure 5, Figure 6 and Figure 7). This fact indicates that many patients postponed dental visits that consequently lead them to seek dental help for a toothache. The authors find this aspect very concerning.

We may assume that the strong decrease in the query amount for the word “dentist” that is highly discernible in our study was caused by a fear of dental visits. The SARS-CoV-2 virus with its rapid transmission made people reluctant to visit public places. With limits on outdoors activities due to the awareness of infection, people postponed their regular dental visits. Smales et al. [22] observed a similar pattern during the SARS epidemic outbreak. It was proved that as the number of diagnosed cases with pneumonia increased, especially in the initial phase of pandemic outbreak, the number of dental visits decreased. In dental studios, according to Peng et al. [23] COVID-19, can be transmitted directly through inhalation or via exposure of nasal, oral or eye mucosa or indirectly via contaminated surfaces [24,25,26]. Triage and screening of dental patients should be carried out, as it is scientifically proved that asymptomatic people can transmit virus to others [27].

It is worth noticing that the lowest RSV search for “dentist” was observed in late March and April (Table 1) that correlates with rigorous policies undertaken by the government regarding lockdown. This is also noticeable in the conducted study with the peak of “stay at home” phrase just after the government applied regulation about the social distancing rules. The figures clearly show the large impact of lockdown on reducing dental visits and amplify differences between countries, which are certainly related to the strategy of combating COVID-19 (Figure 1). The UK and Poland show very similar patterns with high correlations between search queries. It might be explained by the similar time of restrictions. As it was clearly proven in our study, in Sweden, the country with the least stringent prevention and control measures [28], a sharp decline in the interest according to the “dentist” expression was not observed as well as no change or imbalance that could be associated with the pandemic according to the word “toothache” (Figure 7).

In the study, the evidence shows how dental-related queries changed significantly during the ongoing pandemic. It is worth noting that in August, indices for “dentist” demonstrated steady increase in the number of search enquiries. This shows larger interest in dental visits after the time of lockdown and signifies that the number of dental visits during the initial phase of the pandemic was insufficient. It may also forecast the growth in the post-pandemic dental treatment needs.

As shown, during the ongoing pandemic, Google Trends may serve as a powerful tool to give insight into population-level searches for gathering information on how to improve dental services. The relative frequency search query was also conducted during influenza epidemics [29,30]. Constant surveillance and monitoring of Google Trends may help to focus on population treatment needs that have much changed during the COVID-19 pandemic.

The SARS-CoV-2 pandemic has drastically affected dental practices all over the world. The transmission of the virus is relatively high in aerosols; these conditions are met at dental office. According to studies [24,31,32], aerosols, splatters and droplets are produced in many dental procedures. There is a potential hazard to spread cross-infections to dental staff and patients present in the dental office. Presently, as stated in the study by Guo et al. [5]; thirty-eight percent fewer patients visited the dental urgent dental needs at the beginning of the COVID-19 pandemic than before. According to the authors the division of dental problems has also remarkably changed. The dental and oral infection rose from 51.0% to 71.9% when compared to the pre-COVID-19 period. On the other hand, dental trauma decreased from 14.2% to 10.5%. What was highly remarkable was that the non-urgency cases were reduced to three-tenths of pre-COVID-19 dental visits. Bai et al. [33] achieved similar research results with fewer total dental visits in 2020 when compared to 2019. Also, the proportion of patients with acute toothache and infections was higher in 2020 than in 2019, with more drug treatment for acute pulpitis than endodontic treatment and examination consultations. As observed in previous research, as well as in the aforementioned study, the proportions of patients with trauma and non-emergencies had decreased during the pandemic. We may assume that the pandemic has highly affected the number of patients and the structure of oral health problems, usually treated in dental offices. This unprecedented situation should be taken under consideration in order not to narrow patient treatment options; especially with urgent treatment needs.

As stated by the WHO, most oral health conditions can be prevented or may be cured in their early stages. According to the WHO references during the COVID-19 pandemic, effective prevention of oral problems and self-care still remain a high priority [34]. Patients should be given advice through remote consultation or social media channels on how to maintain good oral hygiene. The problem arises because the early stage of an oral health problem is hard to be accurately self-diagnosed by the patient as clinical signs and symptoms may differ. That is why preventive visits were always promoted by health organizations. Patients’ diagnostic needs could have been insufficient during the pandemic period. What is highly alarming is that a significant part of these diagnoses might concern oral cancer, where delayed judgment may decrease survival rate [35]. Currently, health organizations face a great challenge to continue health care programs and encourage people to regular control visits to the dentist to avoid the development of oral diseases, including oral cancer. The fact that in the United States, from 1999 to 2015, the incidence of oral cancer increased by six percent (from 10.9 to 11.6 per 100,000 citizens) may be alarming [2].

As the WHO still recommends limiting the frequency of dental office visits during the pandemic of Covid-19 [8], telemedicine can be used to prevent overcrowding in the healthcare system and at the same time prevent human exposure, reduce costs and time as well as facilitate high-quality care [36,37]. The study conducted in the Department of Otolaryngology, Wuxi Huishan District People’s Hospital in China showed a one-hundred percent increase in the utilization of telehealth during the COVID-19 pandemic, however, this degree of technological adaptation has not been observed nationally [38]. What is important, it does not require medical professional experience and knowledge or using the specialized devices. The patients’ ability to use a mobile phone camera might become a solution or even may give the possibility to communicate with medical professionals by special application, for example WhatsApp (WhatsApp Messenger, WhatsApp Inc., Mountain View, California, USA), and hence inform the clinician about urgent condition [39,40] or improve compliance of patients and oral health status [41]. A special application program for photo sharing images was tested by Incorporating- Beckmann van der Ven et al. [42]. According to the author’s extra- and intraoral photographs, radiographs could be shared with other medical professionals involved in the treatment of a patient via the Internet. Intraoral cameras may improve remote diagnosis and become a part of a regular control visits for patients in the group of oral cancer risk when self-examination can be insufficient. The use of alternative online ways of communication can significantly improve patient-doctor cooperation, however, teledentistry may lead to some difficulties from the technical point of view like in terms of photoshoot [43].

The limitation of this study is that the analyzed data came from the Internet users, which constitutes about 4.57 billion people and stands for 59 percent of the global population [44]. We used only one search engine, but according to [45] it is the most widely used engine with market share estimated at 92.18%. Another limitation of the study is that it does not present absolute search volumes that could lead to more precise studies, however, analysis of data tabulated to the pandemic period led to useful information. Although the number of studies based on Google Trends is growing, there are no prior standards on how to evaluate the literature for new data sources, such as Google Trends [46].

## 5. Conclusions

1. During the increasing search for the word “toothache”, there was no simultaneous response in the larger number of queries for “dentist”. This fact predicts larger dental needs in the post-pandemic period. Strict restrictions undertaken to slowdown the virus transmission have a large impact on dental needs.
2. During this unprecedented situation new strategies and challenges for dental and oral health care system arise. Teledentistry should be taken into account as a possible solution to dental visits.
3. Further studies with online search queries using Google Trends should be performed in order to investigate real time dental treatment needs during the ongoing worldwide pandemic. Especially regarding the paramount challenges that have arisen concerning the prevention and diagnostics of oral cancers.

## Figures and Tables

**Table 1:** Relative search volumes for the whole world, the US, the UK, Poland (PL), Italy (IT) and Sweden (SE).

Week	Toothache World/US/UK/PL/IT/SE	Dentist World/US/UK/PL/IT/SE	Stay at Home World/US/UK/PL/IT/SE
5 January 2020	74/86/67/71/57/86	100/100/98/87/94/78	1/1/3/0/0/2
12 January 2020	73/82/64/64/70/76	97/98/97/92/96/96	1/1/2/0/0/4
19 January 2020	72/83/71/66/59/78	95/94/96/81/92/88	1/1/3/1/2/4
26 January 2020	68/78/59/83/62/96	91/90/93/87/90/87	1/1/3/1/1/3
2 February 2020	73/80/72/69/50/63	92/94/100/89/87/86	1/1/2/1/1/5
9 February 2020	75/78/60/83/56/81	89/90/93/89/89/82	1/1/2/0/0/4
16 February 2020	78/86/67/81/67/57	92/93/93/89/89/79	1/1/2/1/1/6
23 February 2020	69/81/66/82/54/67	91/92/95/76/78/75	1/1/2/0/1/8
1 March 2020	71/78/62/72/50/67	91/91/94/87/80/81	2/1/4/0/10/12
8 March 2020	67/76/58/50/49/70	81/81/89/70/60/72	2/1/10/44/100/51
15 March 2020	72/81/57/83/59/70	75/68/90/62/39/73	27/22/52/60/64/100
22 March 2020	85/97/68/79/66/89	60/50/73/56/38/73	100/100/100/100/21/49
29 March 2020	92/92/88/80/67/73	57/49/61/54/35/68	93/97/52/86/13/39
5 April 2020	96/98/91/74/85/84	54/45/54/51/41/55	46/42/38/59/15/29
12 April 2020	100/97/100/92/71/100	56/49/54/51/48/67	45/44/26/37/11/22
19 April 2020	98/100/91/69/62/94	58/51/55/57/46/56	50/52/25/29/5/18
26 April 2020	95/89/94/88/100/59	57/56/53/58/52/63	62/67/17/29/5/15
3 May 2020	86/85/91/62/64/47	64/67/53/69/72/70	33/35/16/21/3/13
10 May 2020	89/84/82/71/57/41	69/72/61/70/71/68	38/41/17/15/2/13
17 May 2020	87/85/85/84/65/71	75/76/61/72/79/60	20/21/10/12/1/8
24 May 2020	87/82/92/61/59/43	74/75/67/70/75/62	12/12/11/13/1/9
31 May 2020	81/78/74/75/59/45	81/85/70/76/85/66	10/10/7/9/2/7
7 June 2020	84/81/85/79/56/68	85/93/82/74/100/72	6/6/6/6/3/12
14 June 2020	82/85/76/75/59/80	86/94/72/83/94/63	5/5/5/5/2/8
21 June 2020	86/83/81/71/68/93	88/95/75/73/91/68	5/4/5/7/1/10
28 June 2020	83/84/70/76/55/47	85/87/84/83/97/68	6/5/4/8/1/6
5 July 2020	82/89/64/74/58/62	89/95/85/83/92/62	4/3/4/3/1/4
12 July 2020	84/80/71/55/64/78	90/93/87/86/90/65	5/4/4/2/3/4
19 July 2020	76/86/71/86/60/69	89/94/88/94/92/62	4/3/4/4/1/4
26 July 2020	79/84/73/68/59/69	88/94/86/90/95/63	3/2/4/4/2/3
2 August 2020	80/82/68/100/64/66	90/95/86/96/84/72	3/2/6/3/1/3
9 August 2020	77/79/67/71/78/78	90/95/85/84/63/83	2/2/4/2/2/6
16 August 2020	77/74/68/75/50/61	89/92/89/91/69/82	2/2/3/2/2/10
23 August 2020	79/68/67/79/68/62	93/96/97/100/93/100	3/2/4/5/2/11
